# COVID-19 Clinical Features and Outcomes in Elderly Patients during Six Pandemic Waves

**DOI:** 10.3390/jcm11226803

**Published:** 2022-11-17

**Authors:** Roxana Manuela Fericean, Ovidiu Rosca, Cosmin Citu, Diana Manolescu, Vlad Bloanca, Ana-Olivia Toma, Estera Boeriu, Catalin Dumitru, Madhavi Ravulapalli, Vlad Barbos, Cristian Oancea

**Affiliations:** 1 Department XIII, Discipline of Infectious Diseases, “Victor Babes” University of Medicine and Pharmacy Timisoara, Eftimie Murgu Square 2, 300041 Timisoara, Romania; 2 Doctoral School, “Victor Babes” University of Medicine and Pharmacy Timisoara, Eftimie Murgu Square 2, 300041 Timisoara, Romania; 3 Department of Obstetrics and Gynecology, “Victor Babes” University of Medicine and Pharmacy Timisoara, Eftimie Murgu Square 2, 300041 Timisoara, Romania; 4 Department of Radiology, “Victor Babes” University of Medicine and Pharmacy Timisoara, Eftimie Murgu Square 2, 300041 Timisoara, Romania; 5 Department of Plastic Surgery, “Victor Babes” University of Medicine and Pharmacy Timisoara, Eftimie Murgu Square 2, 300041 Timisoara, Romania; 6 Department of Microbiology, “Victor Babes” University of Medicine and Pharmacy Timisoara, Eftimie Murgu Square 2, 300041 Timisoara, Romania; 7 Department of Pediatrics, Discipline of Pediatric Oncology and Hematology, “Victor Babes” University of Medicine and Pharmacy Timisoara, Eftimie Murgu Square 2, 300041 Timisoara, Romania; 8 School of General Medicine, Bhaskar Medical College, Amdapur Road 156-162, Hyderabad 500075, India; 9 Department of Urology, “Victor Babes” University of Medicine and Pharmacy Timisoara, Eftimie Murgu Square 2, 300041 Timisoara, Romania; 10 Center for Research and Innovation in Precision Medicine of Respiratory Diseases, “Victor Babes” University of Medicine and Pharmacy Timisoara, Eftimie Murgu Square 2, 300041 Timisoara, Romania

**Keywords:** COVID-19, SARS-CoV-2 infection, elderly patients, viral epidemiology, infectious diseases

## Abstract

Many elderly patients with severe SARS-CoV-2 infections and COVID-19 infections are admitted to intensive care units. Age was previously identified as an independent risk factor for death and contributed to the greater severity of COVID-19. The elderly may have diminished lung functions, poor reactions to artificial ventilation, and compromised immune systems. However, it is yet uncertain how each pandemic wave and the predominant SARS-CoV-2 strains contribute to varying results and how patient groups such as the elderly are impacted. Comparing six COVID-19 pandemic waves, the objective of this study was to examine the variation in case severity, symptomatology, ICU hospitalizations, and mortality among SARS-CoV-2-infected elderly individuals. The study followed a retrospective design, including 60 eligible patients older than 70 years in each of the six pandemic wave groups, after matching them by the number of comorbidities and gender. SARS-CoV-2 infection during the first, third, and fourth pandemic waves had a significantly higher risk of mortality for hospitalized patients. Confusion and dyspnea at admission were significant risk factors for ICU admission in elderly patients (β = 1.92, respectively β = 3.65). The laboratory parameters identified decreased lymphocytes (β = 2.11), elevated IL-6 (β = 1.96), and procalcitonin (β = 2.46) as the most significant risk factors. The third and fourth COVID-19 waves had considerably more severe infections (31.7% and 26.7%) than the sixth wave (13.3%). Median ICU stay and percentage of patients receiving oxygen support also differed across pandemic waves. However, mortality rates between the six pandemic waves were similar. The average length of hospitalization varied dramatically among the six pandemic waves. Although senior patients are more likely to have worse COVID-19 outcomes after hospitalization, this risk is mitigated by the greater prevalence of comorbidities and frailty among the elderly. The six pandemic waves that were specifically evaluated did not reveal considerably disproportionate variations in terms of patient mortality; however, during the fourth pandemic wave, there were likely more hospitalized patients with severe COVID-19 in Romania. It is probable that certain circulating SARS-CoV-2 strains were more infectious, resulting in an increase in infections and a strain on healthcare systems, which might explain the variations found in our research.

## 1. Introduction

In most individuals, severe acute respiratory syndrome coronavirus 2 (SARS-CoV-2) produces no symptoms or moderate symptoms; it is less lethal than other viral infections, even though 20% of cases, such as those involving elderly persons and those with numerous comorbidities, may develop severe forms and immune system overactivation [1,2,3]. The symptoms of coronavirus disease 2019 (COVID-19) include fever, fatigue, and a dry cough. Interstitial pneumonia, thrombo-embolic events, and acute respiratory distress syndrome (ARDS) are all potential severe symptoms of SARS-CoV-2 infection in at-risk groups such as the elderly [4,5,6,7]. Overactivation of the immune system, triggering a cytokine storm, may produce these effects, although a wide variability of clinical outcomes was hypothesized to exist between circulating SARS-CoV-2 variants [8,9].

All age groups are vulnerable to SARS-CoV-2 infection, and the median hospitalized cohort age is 50–60, with a higher rate of intensive care unit (ICU) admissions and mortality after the age of 65 [10,11,12]. Men are more likely to have SARS-CoV-2 than women of comparable age, and they have a higher prevalence among hospitalized patients needing critical care, which may indicate a difference in severity; although, recent investigations had divergent outcomes [13,14,15]. These symptoms and manifestations have remained all throughout the COVID-19 pandemic development, with almost three years since its onset. However, several investigations show that different SARS-CoV-2 genotypes display different symptomatology and infection severity [16,17].

Many elderly COVID-19 patients with severe infections are admitted to critical care units with elevated inflammatory markers and D-dimer concentrations. The inflammatory cell infiltration in the lungs triggers the cytokine storm syndrome in COVID-19 patients [18,19,20]. Some experts feel that rapid treatment of this cytokine storm in its early stage with immunomodulators, corticosteroids, and cytokine antagonists is an essential component in decreasing mortality rates and reducing ICU hospitalizations [21,22,23]. Aging contributes to the increased severity of COVID-19, and it was previously observed as an independent risk factor for mortality. Elderly individuals may have reduced lung function and a poor response to mechanical ventilation, as well as a weakened immune system [24,25,26].

Although the COVID-19 vaccination campaign was spread worldwide by early 2021, reaching an impressive number of vaccinated patients until 2022, the efficacy of two or even three doses started to become lower as time passed, and the SARS-CoV-2 virus continued suffering different mutations [27,28,29,30,31]. Therefore, it was observed that during different spikes of the pandemic, the spread of infection and its severity changed, encountering more or less hospitalized and severely ill COVID-19 patients. To the best of our knowledge, there are little data on the dynamics of SARS-CoV-2 viral symptoms in elderly patients hospitalized in Romania throughout the last six pandemic waves. Therefore, the purpose of this research was to describe the variance in case severity, symptomatology, ICU hospitalizations, and death among SARS-CoV-2-infected elderly patients in a parallel comparison between six COVID-19 pandemic waves.

## 2. Materials and Methods

### 2.1. Study Design and Ethics

The current research was designed as a retrospective cohort study of hospitalized elderly patients with COVID-19. Patients included in the study were admitted at the Infectious Diseases and Pulmonology Hospital, “Victor Babes”, in the period starting in March 2020 until August 2022. The research protocol was approved on 28 February 2022 by the Ethics Committee of the “Victor Babes” University of Medicine and Pharmacy from Timisoara, Romania, and by the Ethics Committee of the hospital, with approval number 05. This time span covers both the pre- and post-COVID-19 immunization phases. The study took place at the University of Medicine and Pharmacy “Victor Babes” in Timisoara, under the Infectious Disease Department. The goal of this study was to perform retrospective research by gathering information from the paper and electronic hospital records of elderly patients diagnosed with COVID-19 who were hospitalized during the study period.

### 2.2. Inclusion Criteria

A database and patient paper record search were conducted to determine the number of elderly patients admitted to the hospital with a SARS-CoV-2 infection. Patients were included if they matched the following criteria: (1) being older than 70 years; (2) their paper records mentioned the ICD-10 diagnosis code of COVID-19 [32]; (3) the hospitalization occurred due to SARS-CoV-2 infection as the main diagnosis, without other acute conditions at admission; (4) being vaccinated or unvaccinated against SARS-CoV-2; and (5) having a SARS-CoV-2 infection confirmed by a PCR test. According to existing guidelines, the SARS-CoV-2 infection was considered mild, moderate, or severe as follows: (a) presenting to the hospital with a respiratory distress syndrome or respiratory rates higher than 30/min; (b) the finger oxygen saturation measured after 5 min of rest was lower than 93%; (c) PaO2 (the arterial oxygen partial pressure)/FiO2 (the inspired oxygen fraction) ≤ 300 mmHg; and (d) affected lung area on computed tomography (CT) of more than 50% [33,34]. The COVID-19 status was defined by a positive polymerase chain reaction test (PCR) from oropharyngeal and nasal swabs using multiplex RT-PCR [35]. A predefined patient personal form was used to gather demographic, clinical, and outcome data from electronic medical records and identify the patients’ age distribution.

The elderly age of being older than 70 years was considered based on several studies that demonstrated a significantly higher proportion of hospital admissions and changes in mortality rates from SARS-CoV-2 infection after passing this age [36,37]. The acquired patient information was categorized by the pandemic wave at the time of hospital admission as follows: (1) The first wave in Romania was assumed to have occurred between March and October 2020, when Wuhan-Hu-1 (NCBI Reference Sequence: NC 045512.2) was the predominant variation in circulation [38]; (2) the second COVID-19 wave occurred between October 2020 and February 2021, with Clade variants (S: D614G) being the predominant viral strains [39]; (3) the third pandemic wave occurred between February and July 2021, with the Alpha (B.1.1.7) variation being the predominant circulating virus [40]; (4) the fourth COVID-19 wave occurred between July and December of 2021, the Delta (B1617.2) SARS-CoV-2 variant being the most prevalent strain [41,42]; (5) the Omicron viral strain produced the fifth pandemic wave in Romania between December 2021 and March 2022 [43]; (6) lastly, the sixth wave in Romania lasted from March 2022 to July 2022 [44]. For each wave, 60 individuals were included in the study, for a total of 360 elderly adults whose gender and comorbidities were matched with a control group of adults younger than 70 years. It was determined using a convenience sampling method that a total minimum of 139 adult patients younger than 70 years that were hospitalized for SARS-CoV-2 infection is sufficient to provide the statistical power needed for the control group.

### 2.3. Study Variables

The variables considered for analysis were the following: (1) the baseline characteristics of study participants (age, body mass index, gender, area of residence, smoking status, alcohol consumption status, number of comorbidities, COVID-19 vaccination status, and COVID-19 vaccine types); (2) paraclinical findings of the study participants (red blood cell count, white blood cell count, lymphocytes, hemoglobin, hematocrit, alanine aminotransferase, ferritin, erythrocyte sedimentation rate, c-reactive protein, fibrinogen, procalcitonin, d-dimers, interleukin-6, and creatinine; (3) clinical findings and disease outcomes (number of signs and symptoms at admission, clinical signs and symptoms, COVID-19 outcomes, disease severity, duration of hospitalization, ICU admission, viral clearance, SOFA score, duration of ICU stay, intubated patients, oxygen supplementation, and mortality).

### 2.4. Statistical Analysis

The statistical analysis was performed with IBM SPSS v.27 (SPSS. Inc., Chicago, IL, USA), while the significance threshold was set for an alpha value of 0.05. The absolute and relative frequencies of categorical variables were computed and compared using the Chi-square and Fisher’s tests. For the comparison of mean rank differences among nonparametric variables, the Kruskal–Wallis test was used. Parametric continuous variables that followed a normal distribution were compared by mean and standard deviation with the ANOVA test (analysis of variance). A Kaplan-Meier curve was plotted for probabilities of mortality based on the sputum culture results, while the Cox regression identified the hazard ratio for mortality in each of the four groups.

## 3. Results

### 3.1. Normal Weight vs. Overweight Patients

A total of 360 elderly patients (≥70 years) were included for data analysis, in comparison with a control group of 234 adults younger than 70 years, as presented in Table 1. The two study groups were matched by gender proportions and number of comorbidities. The average age of patients in the control group was 60.9 years, compared to 73.6 years in the group of interest. There were no significant differences in their baseline characteristics, except for the body mass index and vaccination status, which were significantly higher in the older patients, compared with the younger adults (25.6 vs. 24.2, *p*-value = 0.002), respectively (15.6% vaccinated patients older than 70 vs. 9.8% in younger adults, *p*-value = 0.044). The most commonly used vaccine was the BNT162b2 in 83.9% of older patients, compared to 60.9% in the control group.

Table 2 presents the paraclinical findings among the two study groups. It was observed that the white blood cell count was significantly higher in the control group compared to the elderly (40.6% of samples outside the normal range vs. 31.9%, *p*-value = 0.031). Similarly, the lymphocyte count was decreased in the elderly (44.4% vs. 54.3%, *p*-value = 0.019). Among the inflammatory markers, CRP, procalcitonin, and IL-6 were statistically significantly more elevated among patients older than 70 years.

The clinical presentation and outcomes in elderly patients hospitalized with COVID-19 and adult patients are presented in Table 3, and it was observed that older patients had significantly fewer symptoms at admission compared to the younger group. Among clinical signs and symptoms, it was observed that patients older than 70 presented with significantly more digestive symptoms (16.4% vs. 8.5%, *p*-value = 0.005), as well as a higher proportion of them having dyspnea and confusion as presenting symptoms (16.9% vs. 10.3%, *p*-value = 0.022), respectively 10.6% vs. 4.7% (*p*-value = 0.011). Contrarily, fever was significantly more often observed among younger patients (75.6% vs. 66.1%, *p*-value = 0.013).

As expected, COVID-19 outcomes were significantly more often affecting the elderly (19.7% vs. 12.4%, *p*-value = 0.019). As a consequence, the mean duration of hospitalization was significantly higher than in younger patients (14.1 days vs. 12.7 days, *p*-value < 0.001). Additionally, the SOFA score and proportion of patients admitted to the ICU were higher in patients older than 70 years (median SOFA score = 6.5 vs. 4.4, *p*-value < 0.001), respectively, 14.7% ICU admission among the elderly patients, compared to 7.7% in the control group (*p*-value = 0.009). The duration of hospitalization and ICU stay were higher in the group of older patients, in correlation with a higher mortality rate of 7.5%, compared to 3.5% among the hospitalized younger patients (*p*-value = 0.039).

### 3.2. Dynamic Comparison of COVID-19 Pandemic Waves

**Figure 1:**
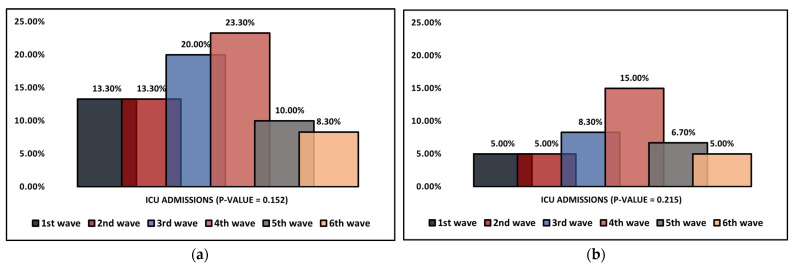
(**a**,**b**) Dynamic comparison of ICU admissions and mortality in elderly patients (≥70 years old) hospitalized with SARS-CoV-2 infection during six COVID-19 pandemic waves.

Table 4 describes the clinical findings of elderly patients hospitalized with SARS-CoV-2 infection over six pandemic waves. It was observed that the COVID-19 severity of hospitalized patients was significantly higher during the third and fourth waves (31.7% and 26.7%, compared with the sixth wave of 13.3% severe infections). The mean duration of hospitalization was observed to vary significantly between the six pandemic waves that were analyzed (*p*-value < 0.001), with the longest hospital stay being observed during the fourth wave (16.4 days), followed by the first wave with an average of 15.3 days. The shortest hospitalization was during the 5th and 6th waves, with 10.3 and 10.5 days, respectively. Other statistically significant differences between the pandemic waves were the median duration of ICU stay and the proportion of patients requiring oxygen supplementation. The longest median duration of hospitalization was during the first wave (7.1 days), followed by the second wave with 6.6 days, while the shortest ICU stay was during the fourth wave (5.2 days, *p*-value = 0.001), as seen in Figure 1a. Despite these differences, the mortality did not significantly change during the six pandemic waves (Figure 1b). Regarding the biological findings measured during the pandemic waves, there was no statistically significant change, as seen in Table 5.

### 3.3. Risk Analysis

**Figure 2:**
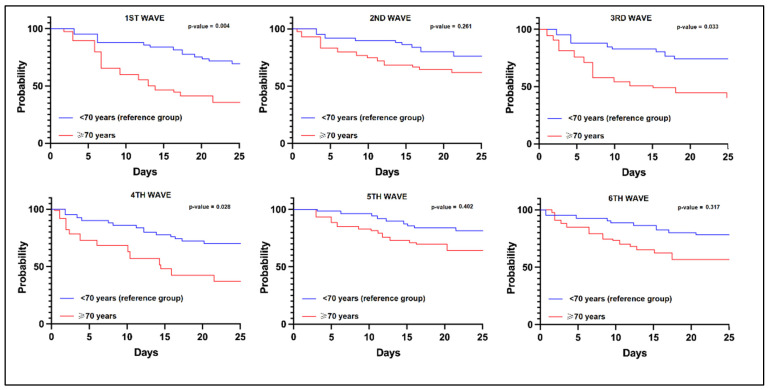
Kaplan-Meier probability analysis for mortality in elderly patients based on the pandemic wave.

The risk analysis for ICU admission in SARS-CoV-2 infected elderly patients was evaluated in Table 6, in comparison with the control group of adults younger than 70. It was observed that the SARS-CoV-2 infection during the first, third, and fourth pandemic waves had a significantly higher risk for mortality, as seen in Figure 2. Among the clinical and paraclinical predictors for ICU admission in the elderly, it was observed that confusion and dyspnea at admission were significant risk factors (β = 1.92 and β = 3.65, respectively). The laboratory parameters identified decreased lymphocytes (β = 2.11), elevated IL-6 (β = 1.96), and procalcitonin (β = 2.46) as the most significant risk factors for ICU admission in the admitted elderly patients.

## 4. Discussion

### 4.1. Literature Findings

In all six waves, fever, cough, and tiredness were the symptoms that occurred most often. Concomitant symptoms that occurred less frequently included a runny nose, headache, and digestive symptoms. Although the first, third, and fourth pandemic waves were observed to bring a significantly higher risk for mortality in the elderly patients hospitalized for COVID-19, the bias risk has to be weighed, considering that the patients admitted to a tertiary clinic and treated were the most difficult cases. Therefore, during peak pandemic waves, it was possible that only the more severe cases were hospitalized. These findings are consistent with previous research [1]; although, we did not evaluate the Pneumonia Severity Index (PSI) score, which was reported to be greater when compared to young and middle-aged adults. It is important to note that among senior patients, the proportion of patients complaining of more severe dyspnea and tachypnea was greater in patients admitted to the ICU, as well as delirium and abdominal discomfort that may accompany cases with a severe evolution [45]. On the other side, constitutional symptoms such as fever and headache were more prevalent in survivors.

Increasing numbers of investigations have shown that older people may have unusual clinical presentations, with fever appearing less commonly in older patients than in younger patients, which was consistent with our findings [46]. Moreover, it appears that delirium and neuropsychiatric symptoms in this patient population are increasing significantly in COVID-19 patients older than 70 years. In a recent meta-analysis of patients with SARS-CoV-2 infection, the prevalence of delirium was almost 30% in those older than 65, compared to less than 15% in the general hospitalized adult population [47], which was associated with an approximately 45% mortality when delirium was present at admission.

Regarding the laboratory findings, it was shown that older patients did not vary significantly from other adults in terms of their WBC, NLR, and procalcitonin levels, although lymphocytes found in the elderly were much lower than in the adult population. On the other hand, the level of CRP found in older individuals was shown to be significantly greater [48]. A comparison of the laboratory findings between the group of elderly patients who survived SARS-CoV-2 infection and those who did not, based on a follow-up period of four weeks, revealed that the number of neutrophils had significantly increased, whereas the number of lymphocytes, monocytes, and platelets had decreased among the deceased patients during the later phases of the infection. However, they did not follow the evolution of laboratory parameters during hospitalization, only at admission. Other findings were that the prothrombin time was considerably extended, coupled with an increase in kidney markers, cardiac markers, and D-dimers [49].

Other studies reported similar results when analyzing hospitalized patients who survived the SARS-CoV-2 infection, identifying that the older population exhibited lower levels of ferritin, procalcitonin, and lymphocytes. It has also been shown that elevated levels of D-dimers, CRP, and a high NLR score are related to a worse prognosis [50], where elevated D-dimers had the best sensitivity and specificity for negative outcomes, followed by CRP levels and NLR score. Other studies that researched the conventionally tested biological markers found an association between LDH and AST with lower pulmonary function, ICU admission, and death [51]. In other investigations, including older individuals with COVID-19, other variables related to mortality, such as frailty, have also been reported. For instance, a recent systematic study that included data from almost one million individuals indicates that frailty and being underweight increased the chance of SARS-CoV-2 infection-associated death by more than five-fold [52].

Prompt identification of COVID-19-related complications is of extreme importance in vulnerable patients such as the elderly. In this case, chest imaging is the most important diagnostic technique for determining pulmonary complications during acute SARS-CoV-2 infection. A bilateral multilobar ground-glass opacification with a peripheral or posterior distribution, primarily in the lower lobes, is one of the typical hallmarks of COVID-19 [53]. In a limited number of instances, particularly those affecting old patients, an atypical first imaging appearance of consolidative opacities superimposed over ground-glass opacity may be seen. It was observed that the elderly had a significantly higher incidence of multiple lobe involvement compared to the younger and middle-aged groups [54].

Although there were more older patients who were vaccinated than younger adults in our cohort, the severity of SARS-CoV-2 infection was much greater among the elderly. It is well advised that the elderly are a high-risk population that should be provided immunization with priority, but it was observed that the antibody response after vaccination was typically lower due to the steady reduction of the immune system with age and the immunological response of neutralizing antibodies after vaccination dropped more abruptly in older individuals than the adult patients with the same vaccines and number of doses [55]. On the contrary, it was also observed that in patients older than 60 years, the rates of severe SARS-CoV-2 infections were significantly lower by almost 20% among those who received a third booster dose of the Pfizer-BioNTech vaccine compared to those who did not receive the third dose [56]. However, current findings show that mRNA-based COVID-19 vaccination boosters are effective against the Omicron variant, but with a lower effect; although, data on the elderly are few [57].

This research identified substantial differences between the six COVID-19 pandemic waves in Romania. Similar studies are few on reporting a complete comparison of each wave with the purpose of determining the variability of SARS-CoV-2 mutations and severity of infections. Another study that took place in Thailand showed that the severity of the third wave represented by the Delta strain was greater than that of prior waves, which is similar to our findings [58]. It is, however, unknown if the difference is attributable to the absence of effective social distancing measures and public health initiatives or a more dangerous mutation of the SARS-CoV-2 virus. On the other hand, the greatest detrimental effects on public health were caused by the first wave. Another study comparing Delta solely and Omicron found a case fatality ratio of 3.4% for Delta and 1.9% for Omicron, indicating a difference of around twice. Consequently, Omicron is less severe than Delta based on these metrics, with the exact severity reduction compared to Delta depending on how the number of infections is assessed [59].

### 4.2. Study Limitations and Strengths

As a first limitation, there is the possibility of human error in the creation of digital data from paper medical records, and the quality of the data that was studied in a retrospective cohort design may have been lower than expected. The second constraint is that there was a very low number of participants in each individual group’s sample, despite the fact that the total number of participants was sufficient to satisfy the statistical power requirements. The third limitation of the current study would be the monocentric design, which can limit the generalization of our findings. A higher rate of COVID-19 complications at admission can occur in patients with multiple comorbidities. To prevent the bias risk of multiple comorbidities in elderly patients, it was opted to include in the study only patients admitted for SARS-CoV-2 infection, excluding those who got infected during their hospital stay for a different diagnosis. Comorbidities such as arterial hypertension and diabetes mellitus were identified more often in some of the study groups, predisposing them to worse outcomes and higher mortality rates, as they seem to be related to a more severe infection [60,61]. Lastly, patients with a previous infection are presumed to develop a stronger immunity against the virus, which could distort the result in this study [62,63]. However, the only way to verify if a patient had a previous SARS-CoV-2 infection or hospitalization for COVID-19 was to check in the hospital’s database if the patient was admitted before, and it was not possible to check if the patient was admitted elsewhere.

## 5. Conclusions

Although elderly patients are likely to have worse COVID-19 outcomes during hospitalization, the risk is weighted by the higher proportion of comorbidities and frailty of the elderly. The six pandemic waves that were particularly analyzed did not show significantly disproportionate differences regarding patient mortality; although, during the fourth wave, there were probably more patients with severe COVID-19 admitted to the hospital. It is likely that some circulating SARS-CoV-2 viral strains were more contagious, causing more infections and creating an overload on the healthcare systems, which might explain the changes observed in our study. Biological parameters also did not vary significantly among the elderly patients during the six waves that were analyzed, although patients older than 70 were more likely to present with dyspnea, confusion, and digestive symptoms, associated with lower lymphocyte levels and higher IL-6 levels. It is, therefore, difficult to diagnose and treat elderly people who have SARS-CoV-2 infection because they are more prone to developing severe clinical consequences from the virus. According to the information that is now available, a customized strategy that targets both the positive and negative consequences of therapy choices need to be made available to older persons. It is imperative that hospitals and residential care facilities that provide long-term care immediately develop appropriate healthcare plans for their older patients. To ensure that COVID-19 patients have access to the most productive therapy choices, fragility must be addressed. Until further advancements can be made in therapy, it is advised that the elderly population be kept isolated from the rest of the community when COVID-19 epidemics occur.

## Figures and Tables

**Table 1:** Comparison of baseline characteristics.

Baseline Characteristics	<70 Years (*n* = 234)	≥70 Years (*n* = 360)	*p*-Value
**Background data**			
Age (years), mean ± SD	60.9 ± 7.8	73.6 ± 8.1	<0.001
BMI, mean ± SD	24.2 ± 5.0	25.6 ± 5.4	0.002
Gender (men)	129 (55.1%)	198 (55.0%)	0.975
Area of residence (urban)	137 (58.5%)	192 (53.3%)	0.211
Smoking	66 (28.2%)	84 (23.3%)	0.181
Alcohol consumer	29 (12.4%)	46 (12.8%)	0.890
**Number of comorbidities**			0.999
0	15 (6.4%)	36 (6.4%)	
1	34 (14.5%)	31 (14.4%)	
2	106 (45.3%)	72 (45.3%)	
≥3	79 (33.8%)	74 (33.9%)	
**COVID-19 vaccination status**			0.044
Yes	23 (9.8%)	56 (15.6%)	
No	211 (90.2%)	304 (84.4%)	
**COVID-19 vaccine**	**(*n* = 23)**	**(*n* = 56)**	0.073
BNT162b2	14 (60.9%)	47 (83.9%)	
mRNA-1273	6 (26.1%)	5 (8.9%)	
Ad26.COV2.S	3 (13.0%)	4 (7.1%)	

Data reported as *n* (%) and calculated using Chi-square test and Fisher’s exact test unless specified differently; BMI—Body Mass Index; BNT162b2—Pfizer BioNTech; mRNA-1273—Moderna; Ad26.COV2.S—Astra Zeneca.

**Table 2:** Paraclinical findings.

Paraclinical Findings	Normal Range	<70 Years (*n* = 234)	≥70 Years (*n* = 360)	*p*-Value
RBC (millions/mm^3^)	4.35–5.65	72 (30.8%)	107 (29.7%)	0.785
WBC (thousands/mm^3^)	4.5–11.0	95 (40.6%)	115 (31.9%)	0.031
Lymphocytes (thousands/mm^3^)	1.0–4.8	127 (54.3%)	160 (44.4%)	0.019
Hemoglobin (g/dL)	13.0–17.0	55 (23.5%)	92 (25.5%)	0.571
Hematocrit (%)	36–48	59 (25.2%)	94 (26.1%)	0.806
ALT (U/L)	7–35	67 (28.6%)	113 (31.4%)	0.475
Ferritin (ng/mL)	20–250	70 (29.9%)	96 (26.7%)	0.388
ESR (mm/h)	0–22	105 (44.9%)	189 (51.1%)	0.069
CRP (mg/L)	0–10	83 (35.5%)	187 (51.9%)	0.001
Fibrinogen (g/L)	2–4	63 (26.9%)	111 (30.8%)	0.306
Procalcitonin (ug/L)	0–0.25	26 (11.1%)	69 (19.2%)	0.008
D-dimers (ng/mL)	<250	23 (9.8%)	54 (15.0%)	0.066
IL-6 (pg/mL)	0–16	53 (22.6%)	127 (35.2%)	0.001
Creatinine (µmol/L)	0.74–1.35	14 (6.0%)	38 (10.6%)	0.054

Data reported as % outside the normal range and calculated using the Chi-square test and Fisher’s exact test unless specified differently; RBC—Red Blood Cells; WBC—White Blood Cells; ESR—Erythrocyte Sedimentation Rate; CRP—C-reactive Protein; IL-6—Interleukin 6; ALT—Alanine Aminotransferase.

**Table 3:** Clinical presentation and outcomes in elderly patients hospitalized with COVID-19 and adult patients.

Variables *	<70 Years (*n* = 234)	≥70 Years (*n* = 360)	*p*-Value
**Number of signs and symptoms at admission**			0.010
0	9 (3.8%)	36 (10.0%)	
1	34 (14.5%)	50 (13.9%)	
2	97 (41.5%)	163 (45.3%)	
≥3	94 (40.2%)	111 (30.8%)	
**Clinical signs and symptoms**			
Digestive symptoms	20 (8.5%)	59 (16.4%)	0.005
Anosmia	42 (17.9%)	55 (15.3%)	0.389
Ageusia	58 (24.8%)	74 (20.6%)	0.225
Fatigue	16 (69.7%)	267 (74.2%)	0.229
Dyspnea	24 (10.3%)	61 (16.9%)	0.022
Confusion	11 (4.7%)	38 (10.6%)	0.011
Headache	23 (9.8%)	44 (12.2%)	0.367
Fever	177 (75.6%)	238 (66.1%)	0.013
Cough	153 (65.4%)	255 (70.8%)	0.161
**COVID-19 Outcomes**			
Severe COVID-19	29 (12.4%)	71 (19.7%)	0.019
Severe imaging features	37 (15.8%)	83 (23.1%)	0.031
Mean duration of hospital stay	12.7 ± 3.3	14.1 ± 4.0	<0.001
Median duration from symptom onset until hospital admission	4.5 (6.5)	3.5 (3.0)	<0.001
Viral clearance	12 (9)	14 (12)	<0.001
ICU admissions	18 (7.7%)	53 (14.7%)	0.009
Median duration from hospital admission to ICU admission	5.0 (7.0)	3.5 (3.0)	<0.001
SOFA score	4.4 (3.1)	6.5 (4.8)	<0.001
Median duration of ICU stay	7.3 (6.6)	5.6 (4.9)	<0.001
Severe in-hospital complications	24 (10.3%)	59 (16.4%)	0.035
Intubation	11 (4.7%)	34 (9.4%)	0.032
Oxygen supplementation	86 (36.8%)	159 (44.2%)	0.072
Mortality	8 (3.4%)	27 (7.5%)	0.039

* Data reported as *n* (%) and calculated using the Chi-square test and Fisher’s exact test unless specified differently; BMI—Body Mass Index; ICU—Intensive Care Unit; SOFA—Sequential Organ Failure Assessment.

**Table 4:** Clinical findings of elderly patients (≥70 years old) hospitalized with SARS-CoV-2 infection stratified by COVID-19 pandemic wave.

Clinical Findings	1st Wave (*n* = 60)	2nd Wave (*n* = 60)	3rd Wave (*n* = 60)	4th Wave (*n* = 60)	5th Wave (*n* = 60)	6th Wave (*n* = 60)	*p*-Value
Severe COVID-19	11 (18.3%)	9 (15.0%)	16 (26.7%)	19 (31.7%)	8 (13.3%)	8 (13.3%)	0.046
Severe imaging features	12 (20.0%)	8 (13.3%)	17 (28.3%)	21 (35.0%)	12 (20.0%)	13 (21.7%)	0.085
Mean duration of hospital stay	15.3 ± 4.0	15.0 ± 4.3	14.1 ± 4.0	16.4 ± 5.2	10.3 ± 3.7	10.5 ± 3.9	<0.001
Median duration from symptom onset until hospital admission	2.0 (2.0)	3.0 (2.5)	3.5 (3.0)	3.0 (2.5)	3.5 (3.0)	4.0 (2.5)	0.122
Viral clearance	15 (11)	14 (13)	16 (14)	15 (12)	14 (11)	14 (12)	0.683
ICU admissions	8 (13.3%)	8 (13.3%)	12 (20.0%)	14 (23.3%)	6 (10.0%)	5 (8.3%)	0.152
Median duration from hospital admission to ICU admission	5.0 (3.0)	4.5 (3.5)	4.0 (3.0)	3.5 (3.0)	3.5 (3.0)	4.0 (3.5)	0.360
SOFA score	5.6 (4.6)	5.8 (4.8)	6.7 (4.9)	6.5 (4.3)	6.8 (5.0)	6.5 (4.8)	0.062
Median duration of ICU stay	7.1 (3.4)	6.6 (3.9)	5.7 (4.0)	5.2 (3.5)	5.4 (3.4)	5.9 (4.2)	0.001
Severe in-hospital complications	7 (11.7%)	8 (13.3%)	13 (21.9%)	15 (25.0%)	7 (11.7%)	9 (15.0%)	0.227
Intubation	4 (6.7%)	5 (8.3%)	7 (11.7%)	10 (16.7%)	4 (6.7%)	4 (6.7%)	0.334
Oxygen supplementation	21 (35.0%)	24 (40.0%)	35 (58.3%)	36 (60.0%)	20 (33.3%)	23 (38.3%)	0.004
Mortality	3 (5.0%)	3 (5.0%)	5 (8.3%)	9 (15.0%)	4 (6.7%)	3 (5.0%)	0.215

Data reported as *n* (%) and calculated using the Chi-square test and Fisher’s exact test unless specified differently; ICU—Intensive Care Unit; SOFA—Sequential Organ Failure Assessment.

**Table 5:** Paraclinical findings of elderly patients (≥70 years old) hospitalized with SARS-CoV-2 infection stratified by COVID-19 pandemic wave.

Paraclinical Findings	Normal Range	1st Wave (*n* = 60)	2nd Wave (*n* = 60)	3rd Wave (*n* = 60)	4th Wave (*n* = 60)	5th Wave (*n* = 60)	6th Wave (*n* = 60)	*p*-Value
RBC (millions/mm^3^)	4.35–5.65	20 (32.8%)	23 (37.7%)	13 (21.3%)	17 (27.9%)	16 (26.2%)	18 (29.5%)	0.454
WBC (thousands/mm^3^)	4.5–11.0	18 (29.5%)	23 (37.7%)	16 (26.2%)	21 (34.4%)	20 (32.8%)	17 (27.9%)	0.750
Lymphocytes (thousands/mm^3^)	1.0–4.8	24 (40.0%)	23 (38.3%)	28 (46.7%)	29 (48.3%)	26 (43.3%)	30 (50.0%)	0.752
Hemoglobin (g/dL)	13.0–17.0	13 (21.3%)	18 (29.5%)	14 (23.0%)	14 (23.0%)	17 (27.9%)	16 (26.2%)	0.889
Hematocrit (%)	36–48	14 (23.0%)	18 (29.5%)	15 (25.0%)	16 (26.7%)	14 (23.0%)	17 (27.9%)	0.949
ALT (U/L)	7–35	17 (27.9%)	19 (31.7%)	20 (33.3%)	27 (45.0%)	17 (27.9%)	13 (21.3%)	0.134
Ferritin (ng/mL)	20-250	15 (25.0%)	12 (20.0%)	16 (26.7%)	17 (27.9%)	12 (20.0%)	14 (23.3%)	0.855
ESR (mm/h)	0–22	32 (53.3%)	30 (50.0%)	28 (46.7%)	36 (60.0%)	35 (58.3%)	28 (46.7%)	0.552
CRP (mg/L)	0–10	29 (48.3%)	31 (51.7%)	27 (45.0%)	38 (63.3%)	32 (53.3%)	30 (50.0%)	0.449
Fibrinogen (g/L)	2–4	19 (31.7%)	18 (29.5%)	15 (25.0%)	17 (27.9%)	20 (33.3%)	21 (34.4%)	0.871
Procalcitonin (ug/L)	0–0.25	11 (18.3%)	9 (15.0%)	13 (21.7%)	15 (25.0%)	15 (25.0%)	9 (15.0%)	0.555
D-dimers (ng/mL)	<250	10 (16.7%)	11 (54.0%)	9 (15.0%)	13 (21.7%)	14 (23.0%)	10 (16.7%)	0.839
IL-6 (pg/mL)	0–16	21 (34.4%)	20 (32.8%)	23 (37.7%)	22 (36.7%)	24 (40.0%)	17 (27.9%)	0.813
Creatinine (µmol/L)	0.74–1.35	8 (13.3%)	8 (13.3%)	6 (10.0%)	9 (15.0%)	4 (6.7%)	5 (8.3%)	0.659

Data reported as % outside the normal range and calculated using the Chi-square test and Fisher’s exact test unless specified differently; RBC—Red Blood Cells; WBC—White Blood Cells; ESR—Erythrocyte Sedimentation Rate; CRP—C-reactive Protein; IL-6—Interleukin 6; ALT—Alanine Aminotransferase.

**Table 6:** Regression analysis for risk of ICU admission in SARS-CoV-2-infected elderly patients.

	β for ICU Admission *	(95% CI of β)	Significance
≥70 years (constant) ^	1.93	1.15–3.66	0.020
**Covariates (predictors)—pandemic waves**			
1st pandemic wave	2.12	1.48–4.20	0.004
2nd pandemic wave	1.59	0.92–2.84	0.261
3rd pandemic wave	2.36	1.28–3.78	0.033
4th pandemic wave	2.04	1.13–4.09	0.028
5th pandemic wave	1.33	0.90–1.83	0.402
6th pandemic wave	1.58	0.87–1.96	0.317
**Covariates (predictors)—clinical and paraclinical**			
Confusion	1.92	1.20–2.47	0.001
Dyspnea	3.65	1.46–5.39	<0.001
Decreased WBC	1.09	0.91–1.43	0.063
Decreased lymphocytes	2.11	1.34–3.06	<0.001
Elevated procalcitonin	2.46	1.52–3.88	<0.001
Elevated IL-6	1.96	1.31–2.95	0.001
Elevated CRP	1.13	0.98–1.42	0.051

* Dependent (response) variable; ^ Estimated risk in univariate analysis; CI—Confidence Interval.

## Data Availability

Data available on request.
